# Skin-inspired, sensory robots for electronic implants

**DOI:** 10.21203/rs.3.rs-3665801/v1

**Published:** 2023-12-22

**Authors:** Wubin Bai, Lin Zhang, Sicheng Xing, Haifeng Yin, Hannah Weisbecker, Hiep Thanh Tran, Ziheng Guo, Tianhong Han, Yihang Wang, Yihan Liu, Yizhang Wu, Wanrong Xie, Chuqi Huang, Wei Luo, Michael Demaesschalck, Collin McKinney, Samuel Hankley, Amber Huang, Brynn Brusseau, Jett Messenger, Yici Zou

**Affiliations:** University of North Carolina, Chapel Hill; University of North Carolina; University of North Carolina at Chapel Hill; UNC at Chapel Hill; University of North Carolina at Chapel Hill; UNC at Chapel Hill; University of North Carolina; UNC at Chapel Hill; University of North Carolina; Department of Applied Physical Sciences, The University of North Carolina at Chapel Hill; University of North Carolina, Chapel Hill; UNC at Chapel Hill; UNC at Chapel Hill; UNC at Chapel Hill; University of North Carolina at Chapel Hill; UNC at Chapel Hill; UNC at Chapel Hill; UNC at Chapel Hill; Purdue University; UNC at Chapel Hill

## Abstract

Living organisms with motor and sensor units integrated seamlessly demonstrate effective adaptation to dynamically changing environments. Drawing inspiration from cohesive integration of skeletal muscles and sensory skins in these organisms, we present a design strategy of soft robots, primarily consisting of an electronic skin (e-skin) and an artificial muscle, that naturally couples multifunctional sensing and on-demand actuation in a biocompatible platform. We introduce an *in situ* solution-based method to create an e-skin layer with diverse sensing materials (e.g., silver nanowires, reduced graphene oxide, MXene, and conductive polymers) incorporated within a polymer matrix (e.g., polyimide), imitating complex skin receptors to perceive various stimuli. Biomimicry designs (e.g., starfish and chiral seedpods) of the robots enable various motions (e.g., bending, expanding, and twisting) on demand and realize good fixation and stress-free contact with tissues. Furthermore, integration of a battery-free wireless module into these robots enables operation and communication without tethering, thus enhancing the safety and biocompatibility as minimally invasive implants. Demonstrations range from a robotic cuff encircling a blood vessel for detecting blood pressure, to a robotic gripper holding onto a bladder for tracking bladder volume, an ingestible robot residing inside stomach for pH sensing and on-site drug delivery, and a robotic patch wrapping onto a beating heart for quantifying cardiac contractility, temperature and applying cardiac pacing, highlighting the application versatilities and potentials of the nature-inspired soft robots. Our designs establish a universal strategy with a broad range of sensing and responsive materials, to form integrated soft robots for medical technology and beyond.

## Introduction

The dynamically changing environments drive living organisms to evolve toward inseparable integration of motor and sensor functions [1–3]. Especially, coherent integration between skeletal muscles and sensory skins enables their rational and well-organized cooperation orchestrated by neural systems to execute perceptive actions with intelligence. A diverse variety of receptors (mechano, thermal, pain, and others) embedded in the soft skin gathers and encodes tactile data, which not only guides muscular motions to the optimum but also interprets the environment for enhanced awareness and cognition [4–6]. Such motor-sensor integration established in the biological systems inspires development of intelligent robotic systems mimicking skin softness to safely explore and interact with dynamic, unstructured, and often uncertain environments, particularly when robots interface with biological tissues and organs to enable precision therapeutics [7–10]. However, existing robots often lack a seamless integration among actuators, sensors, and controllers, that naturally preserve physical softness and biocompatibility [11,12].

Creating such nature-inspired somatosensory soft robots as implants holds promising potential to innovate medical technology, especially in surgery, diagnosis, drug delivery, prostheses, artificial organs, and tissue-mimicking active simulators for rehabilitation [13–17]. Conceptually distinct types of soft robotic implants take the form of shape-morphing and functionalization, are capable of compliance matching biological tissues, retrieving their functional signature, and offering therapeutic treatments [10,13,14,18–21]. For example, an integrated bladder system consisting of interdigitated capacitive sensors capable of continuous bladder volume detection, and a shape memory alloy-based actuator with strong emptying force for urine voiding, allows for real-time bladder control [18]. A soft gripper based on a shape memory polymer with integration of silver nanowires and a crack-based strain sensor enables conformable contact with a carotid of swine model and measuring its blood pressure [19]. Taking inspiration from the ventricular teeth of hookworm, thera-grippers made of a metal-polymer hybrid actuator and a drug-eluting patch can latch onto the mucosal tissue inside gastrointestinal GI lumen and extend drug release [20]. The combination of sensing and actuation not only enhances diagnostic and/or therapeutic precision for implants via dynamically modulating the structural interface to targeted tissues, but also enables possibility to become artificial organs that offer both needed structural transformation and physiological functions (e.g. electrical signaling, and hormone secretion) [17,20,22]. Despite the promising progress in soft robotic implants, grand challenges remain in designing materials and manufacturing technologies, to leverage multi-faceted requirements of device performance, including compliant mechanics to match tissue softness, biocompatibility of constituent materials to ensure implantation safety, structural adaptability to prolong device longevity, and biomimicry to enhance device functionality [11,12,18,23].

**Figure 1:**
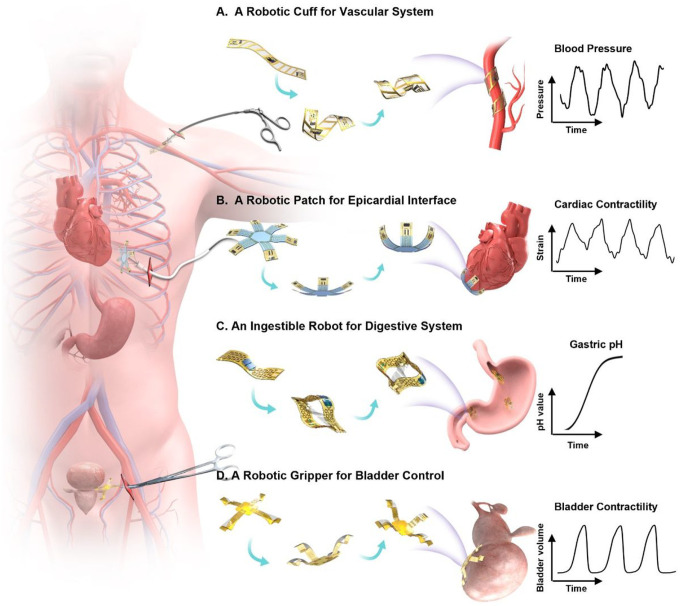
Schematic illustration showing nature-inspired sensory robots as minimally invasive smart implants for diagnosis, stimulation, and drug delivery. **(A)** A robotic cuff for vascular system. The twisting motion provides physical enclosing around a blood vessel for precise detection of blood pressure and structural support. (**B**) A robotic patch for epicardial interface. The gripping motion enables gentle contact with a beating heart without residual straining, to provide real-time quantification of cardiac contractility and temperature, and apply coordinated electrical stimulation for cardiac pacing. **(C)** An ingestible robot for digestive system. This structural transition from the shape of a miniaturized pill to a 3D expanded hoop enables extended stay inside stomach to provide both pH sensing and drug delivery. **(D)** A robotic gripper for bladder control. The adaptive motion of gripping onto a bladder provides precise tracking of bladder volume and targeted stimulation to sacral nerve for treating urological disorders.

Here we present concepts and device designs to achieve untethered, soft robots that follow a biomimicry integration of actuators, sensors, and stimulators, to enable structural adaption and dynamic reconfiguration that minimize tissue damage during implant deployment, release stress at biotic-abiotic interface, increase biocompatibility, and enhance device multi-modal performance with spatiotemporal precision (Fig. 1 and **Fig. S1**). Demonstrated examples of utilizing such nature-inspired robots include: i) a robotic gripper that wraps around a bladder to enable coordinated, closed-loop operation of bladder-volume evaluation and electrical stimulation to treat overactive bladder, ii) a robotic cuff that can enclose around a blood vessel for measure blood flow and pressure, iii) an ingestible robot that can expand when arriving in a stomach for prolonged monitoring and drug delivery, and iv) a robotic patch that can actively grasp and release a beating heart for epicardial sensing and pacing, which collectively highlight potential impacts of the nature-inspired robotic designs that naturally integrate actuation, sensing and stimulation within a coherent entity as next-generation electronic implant with physical intelligence.

The soft robots primarily consist of two integrated, functional layers that emulate relations between sensory skin and underlying muscles. Specifically, one layer represents an electronic skin (e-skin), that is made of functional nanocomposites predominantly based on an *in situ* solution-based fabrication approach. The other layer represents an artificial muscle, that is based on poly(*N*-isopropylacrylamide) (PNIPAM) hydrogel, which can reversibly contract and relax upon activation trigger. Significantly, hydrogels, known for their exceptional softness, low activation temperature and nonfibrotic biocompatibility, are generally preferred over other stimuli-responsive materials in implantable applications [24–27]. The bilayer design composed of the e-skin and artificial muscle represents a heterogeneous configuration with a variety of responsiveness upon exposure to environmental stimuli, which orchestrates its robotic motion. Our *in situ* solution-based method successfully embeds multiple sensing materials (e.g., silver nanowires ~ AgNWs, reduced graphene oxide ~ RGO, MXene and poly(3,4-ethylenedioxythiophene) polystyrene sulfonate ~ PEDOT:PSS) into a polymer matrix (e.g., polyimide ~ PI, and polydimethylsiloxane ~ PDMS), enabling the e-skin a versatile platform that highly mimics the skin with complex receptors, and accurately detects the external signals. These functionalities encompass touch, pressure, temperature, and chemical sensing, surpassing the integrative complexity and heterogeneity achievable with 3D printing or other conventional approaches [28–30]. Moreover, inspired by nature (e.g., starfish and chiral seedpods), we can vary designs of the soft robots, enabling various motions (e.g., bending, expanding, and twisting), and corresponding 3D deformed configurations. In addition, the soft robots allow both on-demand transformation and local-region actuation via embedded control circuits, which further increases structural versatility and capability. Moreover, the soft robots can move, sense, and communicate in a wireless closed-loop fashion via integration of control module and data analytics, enabling minimally invasive operations with safe and stable access to enclosed small spaces inside human body.

## Results

### Nature-inspired multi-modal sensory soft robot

**Figure 2:**
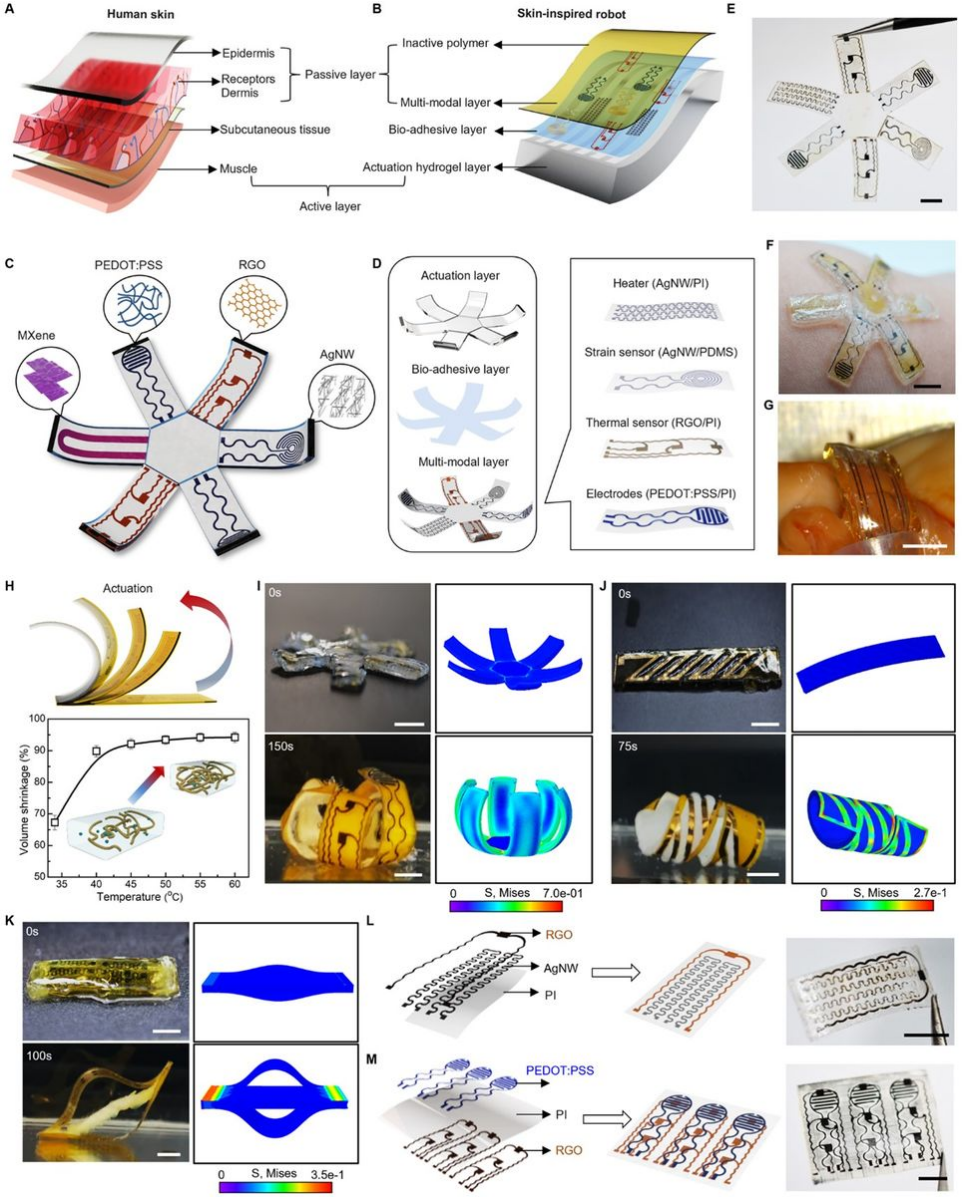
Multi-materials Integration for Multi-modal Sensory Soft Robot. **(A)** Schematic illustration of epidermis-dermis-muscle structure of skin. **(B)** Nature-inspired structure of soft robot from skin. **(C)** Conceptual illustration of the integrated multi-modal sensory soft robot with distinct nanocomposite sensors functionalized into each arm. **(D)** Schematic illustration of a multi-modal sensory soft robot with a nature-inspired starfish design. Left: schematic illustration on an exploded view of the robot, highlighting 3 primary constituent layers, including a flexible multi-modal layer, a bio-adhesive layer, and an actuation hydrogel layer. Right: Schematic illustration highlighting the multi-material integration within the multi-modal layer including: (i) A nanocomposite of silver nanowires (AgNWs) and polyimide (PI) as a flexible heater; (ii) A nanocomposite of AgNWs and PDMS as a strain sensor; (iii), A nanocomposite of reduced graphene oxide (RGO) and PI as a temperature sensor; (iv), A nanocomposite of poly(3,4-ethylenedioxythiophene) polystyrene sulfonate (PEDOT:PSS) and PI as sensing and stimulation electrodes. **(E)** Optical image of the fabricated flexible multi-modal e-skin functionalized with six nanocomposite sensors. **(F&G)** Optical image showing conformal attachment of the soft sensory robot onto human skin **(F)** and porcine tissue **(G)** with high mechanical compliance. **(H)** Top: Schematic illustration showing a temperature-responsive bending behavior resulted from a bilayer design using poly(N-isopropylacrylamide) (PNIPAM) and polyimide-based nanocomposite. Bottom: The volumetric shrinkage of PNIPAM at various temperatures during heating process. **(I-K)** Optical images and corresponding finite element modeling of structural reconfiguration of soft sensory robots upon a thermal trigger. The colors in the legend indicate the magnitude of the von Mises stress in the configurations. (I) Biomimicry soft gripper undergoes enclosing motion upon heating at 40 °C. **(J)** A soft sensory robot inspired by chiral seedpods undergoes a helix reversal upon heating at 40 °C. **(K)** A soft robotic pill based on an anchored hydrogel/nanocomposite tri-layer structure undergoes expanding motion upon heating at 40 °C. **(L&M)** Schematic illustration and optical images of representative examples of anisotropic integration of various functional materials into a polymeric matrix to form a multi-modal sensing system using a solution-based approach. **(L)** A RGO/PI-based temperature sensor and an AgNW/PI-based heater integrated on the same side of a polyimide layer. **(M)** PEDOT:PSS/PI-based electrodes and RGO/PI temperature sensors integrated on the two opposite sides of a polyimide layer, respectively. Scale bars, 5mm.

The integrated architecture between the skin and skeletal muscles enables safe and closed-loop interactions with surrounding environment, bringing a feeling of touch and acting of motion seamlessly together in space and time [31–33]. A feature of particular interest in skin is the mechanoreceptors localized at the interface between the epidermis and dermis of skin, which are responsible for detecting a variety of mechanical stimuli, including the fast-adapting (FA) receptors that respond to dynamic forces and slow-adapting (SA) receptors that respond to static pressures (Fig. 2A) [34–36]. By mimicking the hierarchical architectures of skin and muscle with associated biological functions, our soft robots integrate multi-electronic modules and thermally actuatable hydrogels, realizing both receptor-like sensing functions to detect various stimuli, and on-demand muscle-like contraction to generate physically adaptive motion, from a single integrated platform, endowing soft robots with intelligence in navigating through real-world environments autonomously (Fig. 2B). Figure 2C shows a fabricated multi-modal sensory soft robot with geometry emulating a starfish. The robot consists of three primary layers: a flexible nanocomposite layer as a multi-modal electronic-skin (e-skin) embedded with distinct sensors (strain, pressure, pH, and temperature) and stimulators (thermal and electrical), a thermally responsive hydrogel layer as an artificial muscle generating actuation force, and a thin bio-adhesive layer as a cushioning medium to form interfacial binding between the e-skin and artificial muscle.

Our approach for fabricating the flexible nanocomposite layer can be generally applicable to a wide variety of soft materials and nanomaterials heterogeneously composited within a single matrix material, which enables the potential to form highly integrated systems with a broad range of sensors and stimulators. Specifically, here we demonstrate (1) a thermal sensor made of a nanocomposite of reduced graphene oxide (RGO) and polyimide (PI), (2) a strain sensor made of a nanocomposite of silver nanowires (AgNWs) and polydimethylsiloxane (PDMS), (3) sensing and stimulation electrodes made of a nanocomposite of poly(3,4-ethylenedioxythiophene): polystyrene sulfonate (PEDOT:PSS) and PI, and (4) a thermal heater made of a nanocomposite of AgNWs and PI. The flexibility and versatility of this strategy allow the as-fabricated functional units to be easily integrated into each arm of the starfish robot in a monolithic fashion, as illustrated in Fig. 2D, realizing a sensory skin for the robot to enable environmental awareness. Figure 2E and **Fig. S2B** display an example of an ultrathin multi-modal e-skin equipped with six nanocomposite sensors (The detailed fabrication process appears in **Fig. S2A**.). The e-skin is further encapsulated with a parylene layer (thickness ~ 2 μm) to enhance its durability during prolonged applications (**Fig. S3A**, and **Supplementary Note S1**) [37,38]. This is subsequently attached onto a piece of predesigned thermally responsive PNIPAM hydrogel that serves as an artificial muscle for the soft robot (**Fig. S3B**). The PNIPAM hydrogel can undergo a notable volumetric change about 90% as the temperature change from 23 °C to 40 °C, and thus provide excellent actuation in biological environment (**Fig. S4A–C**) [39,40]. The as-fabricated soft robot can form a highly conformal interface with diverse biological surfaces, indicating its inherent mechanical softness and high biocompatibility (Fig. 2F, Fig. 2G, and **Fig. S2C–E**). At an elevated temperature (40 °C), the robot consisting of the stimuli-responsive artificial muscle (PNIPAM layer) and non-stimuli-responsive e-skin (multi-modal layer) tends to bend toward the muscle side on the basis of asymmetrical responsive properties (Fig. 2H, **Fig. S2F** and **Fig. S4D–F**) [41,42].

Figure 2I, **Fig. S2F**, and **Supplementary Movie S1** provide an example of a robotic starfish reversibly closing and opening its rays under a temperature shift between 23 °C and 40 °C. Furthermore, such multi-layer integration allows a diverse collection of robotic designs that undergo various types of actuation. **Fig. S5**&**6** and **Supplementary Movie S2** highlight soft sensory robots featuring starfish-like structures with an arbitrary number of rays (e.g., four and six) and a fishbone-like structure with four pins. In addition, tuning the design layout of the multi-layer structure can yield complex deformation and structural reconfiguration beyond simple bending motion. Here, inspired by the self-twisting of chiral seedpods, we constructed a geometry with parallel strips in the e-skin layer (Additional details appear in **Fig. S7A** and **Fig. S8A**.). Upon thermal stimulation, concentrated internal stresses at the bonding areas are built simultaneously, leading to local saddle-like curvature and twisting motion of the integrated robotic systems (Fig. 2J, **Fig. S7B, Fig. S8B**, and **Supplementary Movie S3**). We also develop a soft robotic pill based on an anchored hydrogel/nanocomposite tri-layer structure, where a hydrogel-muscle layer is surrounded by two separate e-skin layers that bond to the muscle layer at the edges (**Fig. S9A** and **Fig. S10A** show the detailed structure design.). Upon thermal actuation, the pill can self-expand into a 3D ring shape, since the contraction of the muscle layer drives the e-skin layers to buckle out of the plane, as shown in Fig. 2K, **Fig. S9B**, **Fig. S10B**, and **Supplementary Movie S4**. Furthermore, compared with a make-and-transfer method, our *in situ* solution-based fabrication approach for full integration of sensors into the e-skin layer, in which the sensing material and passive polymer-based skin are structured monolithically, endows the as-fabricated e-skin as a versatile platform that can be constructed using a broad range of functional nanomaterials hybridized with a polymeric matrix to form a multi-modal sensing system, emulating the skin with complex somatosensory system, where various mechanoreceptors and thermoreceptors distributed in the epidermal and dermal layers enable the spatiotemporal recognition of the magnitude and location of touch and temperature stimuli [43,44]. **Fig. S11** provides a representative example of *in situ* integration of AgNW/PDMS-based strain sensor, in which the strain sensor has a good response to the stretching. Figure 2L shows the proposed solution-based approach enables a freestanding PI film to integrate multi-functional modalities including an RGO/PI-based temperature sensor and an AgNW/PI-based heater (**Fig. S12A**), endowing soft robots with both thermal sensing and stimulation. Moreover, Fig. 2M displays a more complicated integration paradigm with multi-layer stacking, where different electronic components (e.g., PEDOT:PSS/PI-based conductive electrodes and RGO/PI temperature sensors) can be distributed in different layers of the e-skin to achieve functional versatility simultaneously and with high proximity (**Fig. S12B**). The X-ray photoelectron spectroscopy (XPS) characterization on the e-skin layers reveals the chemistry of the composite assembled at nanoscale with the active nanomaterials and a polymer matrix. The detailed chemical composition analysis of the nanocomposite appears in **Figs. S13–S15** and **Supplementary Note S2** [45–49]. These functionalities would be of great value for soft robots that seek to achieve multifunctionality and local sensing capabilities approaching skin.

### On-demand robotic actuation with spatiotemporal control

**Figure 3:**
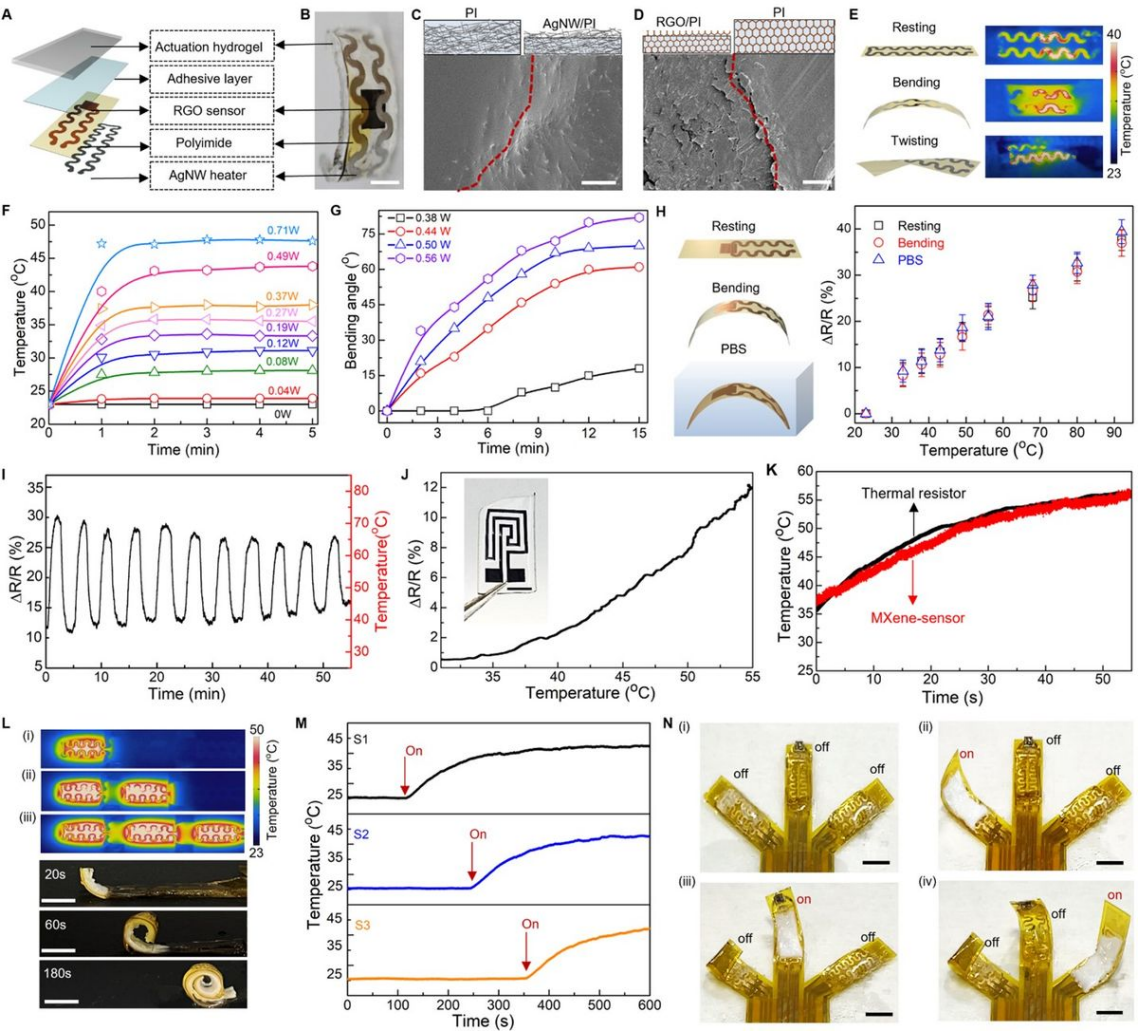
Anisotropic integration of nanocomposites for on-demand robotic actuation with spatiotemporal control. **(A&B)** Schematic illustration showing an exploded view **(A)** and optical image **(B)** of a soft robotic arm comprising a PNIPAM hydrogel layer, a thermal sensor based on RGO/PI nanocomposite, and an actuation heater based on AgNW/PI nanocomposite. The hydrogel is bonded onto the multi-functional nanocomposite layer via n-butyl cyanoacrylate adhesive. Joule heating generated by the electrical heater based on AgNW/PI nanocomposite triggers the robotic actuation of the PNIPAM hydrogel to bend the robotic arm. Scale bar, 5mm. **(C&D)** SEM images of nanocomposite films used in constructing the sensory robots. Here, deep reactive ion etching (DRIE) of partial regions of the nanocomposite films reveals the anisotropic integration within the films where the pristine PI regions form clear boundaries with AgNW/PI regions **(C)**, and RGO/PI regions **(D)**, respectively. In the nanocomposite regions, nearly all the AgNWs or RGO are uniformly dispersed inside the PI matrix, as AgNWs and RGO have higher surface-free energy than that of PI, leading to excellent wetting behavior and high binding strength inside PI. Scale bars, **(C)** 1.5 μm, **(D)** 10 μm. **(E)** Infrared thermograph of an AgNW/PI-based heater undergoing bending and twisting motions. The nanocomposite heater exhibits consistent heating performance after 1000 bending and twisting cycles **(Fig. S18). (F)** Surface temperature of the AgNW/PI-based heater as a function of the input electric power. Notably, the AgNW/PI nanocomposite heater can function under relatively low input electric power. **(G)** The resultant bending angle of a soft robotic arm as a function of the input electric power. **(H)** Resistive response at various temperatures ranging from 23 °C to 92 °C, for the RGO/PI-based thermal sensor undergoing bending and twisting motions, and immersed in a solution of PBS. **(I)** Static cycling test of the RGO/PI-based thermal sensor. **(J)** Resistive response of the MXene- based thermal sensor for temperature ranging from 23 °C to 55 °C. **(K)** Temperature measurement on the MXene/PI thermal sensor and a commercial thermal resistor (ERT-J0ET102H ). **(L)** Stepwise actuation of coiling via sequential thermal stimulus. **Fig. S25** describes the fabrication strategy of integrating AgNW/PI-based heaters and RGO/PI-based thermal sensors into a single flexible film compatible to serve as part of the bilayer design of the sensory robot. Top: Infrared thermograph of AgNW/PI-based heaters via sequential power input. Bottom: Optical images showing the corresponding structural change of the soft robotic probe from a flat state to a coiled state upon sequential thermal activation. Scale bars, 10mm. **(M)** The integrated RGO/PI-based thermal sensors enable temperature measurement of localized regions to realize proprioceptive sensing. **(N)** Optical images demonstrating the on-demand motion control of a three-arm soft robotic gripper via sequentially programming input power. Scale bars, 5mm.

Programmable stimuli-responsive soft robotic systems capable of working in enclosed or confined spaces and adapting functions under changing situations hold great promise as next-generation medical robots [50,51]. The realization of versatile morphing modes through local-actuation control is crucial for enhancing on-demand actuation. Here, we develop soft robots with cognitive capabilities via unifying programmable actuation and *in situ* sensing. Figure 3A shows a sensory robotic arm primarily consisting of a PNIPAM-hydrogel-based muscle layer and a multi-modal e-skin layer that is designed with an AgNW/PI nanocomposite actuation heater and an RGO/PI nanocomposite thermal sensor. **Fig. S16A** shows layer-by-layer stacking as a simple and effective approach for fabricating the e-skin. **Fig. S16B** and Fig. 3B demonstrate the multifunctional nanocomposite film is highly flexible and can be tightly bonded onto the hydrogel layer via n-butyl cyanoacrylate adhesive. When the liquid PI is cast onto the nanomaterial film (e.g., AgNWs and RGO), the liquid PI penetrates into the interconnected pores of the three-dimensional (3D) network, owing to the low viscosity and low surface energy of the liquid PI [52]. The scanning electron microscope (SEM) images (Fig. 3C&D, **Fig. S17A–C**, and **Fig. S23A–C**) present that the curing process fully buries all the nanomaterials inside the PI matrix without generating observable voids. This prevents separation of the nanomaterials from the polymeric matrix, thus minimizing untended side effects to the neighboring tissues. The Fourier-transform infrared spectroscopy (FTIR) and X-ray powder diffraction (XRD) results (**Fig. S17D**&**E**, **Fig. S23D**&**E**, and **Supplementary Note S3**) also confirm the chemical structure of AgNW/PI and RGO/PI nanocomposites suggesting a successful fabrication of high-quality nanocomposite of functional materials and polymer matrix [53–56]. Figure 3E demonstrates that the resultant AgNW/PI nanocomposite conductor is highly conductive, twistable, and bendable, and thus used as a highly flexible heater for electrothermal actuation. Thermal images in Fig. 3E and **Fig. S18A** show the infrared thermograph of a Joule-heated e-skin in the resting state and under both bending and twisting conditions. The electrical heater distributed inside the e-skin exhibits a stable and uniform temperature distribution without degradation of the temperature level at the deformed points, ensuring effective thermal transport from the heater to the hydrogel-based muscle layer. Figure 3F and **Fig. S18B**&**C** depict the transient electrothermal response of the flexible heater applied by various powers in an ex vivo environment. The saturation temperature of AgNW/PI nanocomposite heater increases with the supplied power as more Joule heat is generated, and the lower critical solution temperature (LCST) of PNIPAM hydrogel at 34°C can be obtained at low input power ( < ~ 1W) (**Fig. S19A**). As shown in **Fig. S18C**, the flexible electrical heater exhibits steady heating and cooling cycles, indicating high repeatability and remarkable heating stability using AgNW/PI nanocomposite. Figure 3G and **Fig. S19B** show varying the amount of electric power applied to the heater effectively modulates the resultant bending angle *θ* (as defined in **Fig. S18D**) of the robotic arm, thus realizing precise, on-demand access to intermediate morphologies along the bending pathway. We also investigate the bending behavior of the soft robotic finger in a tissue culture incubator (37 °C) mimicking *in vivo* condition (**Fig. S20**). As expected, a lower electrical power is found to be sufficient for achieving a bending performance similar to that obtained in an *in vitro* condition (ambient condition). We further evaluated the mechanical force generated by the soft robotic finger under various input powers. **Fig. S19C** shows that the static force exhibits a noticeable increase with rising temperature. At a temperature of 40 °C, the force reaches a maximum of 32 mN. Additionally, it is observed that the generated force remains consistent throughout 40 cycles of alternating power on and off (0.35 W), indicating the robust reversibility of the soft robot (**Fig. S19D**). When compared to similar hydrogel-based soft actuators, our design consistently achieves a relatively high output force, as shown in **Table S1** [57–64]. Furthermore, **Fig. S21** demonstrates local activation of a series of motions via integrating multiple AgNW/PI-nanocomposite heaters into a single e-skin platform and precisely commanding them to generate local heating, further highlighting the advantage of our *in situ* solution-based fabrication approach, with high design versatility, simplicity, integrability, and low cost. To exemplify this versatility, we have also successfully fabricated MXene-based sensors, as illustrated in Fig. 3J, Fig. 3K and **Fig. S22**. MXene-based materials possess inherent biocompatibility, rendering them ideally suited for incorporation into implantable biomedical devices without eliciting adverse reactions or compromising the surrounding biological environment [65,66]. The ability to precisely identify the real-time information of temperature during the muscle motion using an RGO/PI-nanocomposite temperature sensor forms a closed feedback loop for the robotic control. The *in situ* solution processing ensures RGO network is uniformly distributed in PI matrix to provide precise pattern registration with good electrical conductivity (~ 400 S/m) and stable performance in aspects of electrical linearity, sensitivity, and repeatability. Figure 3H illustrates the resistive change in a relatively linear relation with temperature for the RGO/PI thermal sensor. The temperature coefficient of the resistance (TCR) of the RGO/PI thermal sensor is > 0.5%/°C, featuring its high thermal sensitivity. On the other hand, the RGO/PI-based thermal sensor exhibits a stable performance after 1000 bending cycles, and even after immersing in PBS solution. Figure 3I and **Fig. S23F** show performance of the thermal sensor in response to cycles of temperature rise and drop, indicating good sensing stability. In addition, the *in situ* solution process allows fabrication of thin films of AgNW/PI nanocomposite as thermal sensors. **Fig. S24A** shows the AgNW/PI thermal sensor exhibits a linear relationship between the resistance and temperature with sensitivity ~ 0.4%/°C over the range of 23–80 °C. Notably, the sensor showcases minimal performance degradation even when subjected to bending and immersion in PBS. Moreover, to evaluate the biostability of the sensor, it is immersed in a PBS solution at 37 °C simulating an *in vivo* condition. **Fig. S24B** indicates the performance of the AgNW-based nanocomposite sensor with outstanding bio-stability over a week. **Fig. S24C** demonstrates good sensing stability of the AgNW/PI-nanocomposite thermal sensor under cyclic temperature test between 25 and 40 °C. In addition, **Fig. S23G** and **Fig. S24D** show consistent measurements of both RGO/PI and AgNW/PI nanocomposite sensing performance in comparison with a commercial thermal resistor (ERT-J0ET102H), indicating excellent sensing accuracy.

Well-controlled shape morphability with high spatiotemporal resolution is essential for soft robots to execute complicated tasks safely in biological environments [67,68]. Here, the integration of e-skin and artificial muscle allows the nature-inspired soft robots to realize motions of local regions independently, which collectively can lead to a wide range of shape morphability. Figure 3L demonstrates the locally controlled morphability with a soft robotic finger, of which the e-skin includes a series of AgNW/PI-based heaters for localized thermal activation, and RGO/PI-based thermal sensors for temperature monitoring (**Fig. S25A**). As shown in Fig. 3L and **Fig. S25B**, the robotic finger undergoes a stepwise self-coiling motion via a sequential stimulus and perceives its local temperature simultaneously by the embedded thermal sensors (Fig. 3M). More complex actuation modes or configurations are accessible owing to the combined ease of processing and ability to introduce multifunctional units. Figure 3N presents a three-arm soft robotic gripper where each arm contains various types of sensors, including an optical sensor, a thermal sensor, and a strain sensor, respectively, and an electrical heater (**Fig. S26A**), to enable independent control of motion for each arm, thus allowing on-demand gripping and interaction with targeted objects (**Supplementary Movie S5**). Moreover, the soft robotic systems can be structurally tailored to support a range of motions. For example, **Fig. S26B**&**C** highlights a soft starfish-like robot with four sensory arms, and a soft robotic cuff with optoelectronic sensors, respectively. The electrothermal stimulus along with distributed sensing capabilities enables programmed actuation not only on demand but also regulated simultaneously by the sensing feedback (e.g., **Supplementary Movie S6**).

### Wireless sensing and actuation of soft sensory robot

Wireless operation of actuation as well as therapeutic and/or diagnostic functions is essential for implantable robots to minimize tissue damage and implant infection [67,69–71]. However, existing schemes for wireless operation of robots mostly require sophisticated circuitry for energy harvesting and storage, which may introduce non-negligible heat (~ 80 °C), unfavored space occupation, system complexity, limited lifetimes, and high cost [72,73]. To overcome the preceding challenges of actuation/sensing integration in soft implantable robots, we report a nature-inspired soft robot as a representative example to demonstrate remote, battery-free operation and communication in both sensing and actuation.

**Figure 4:**
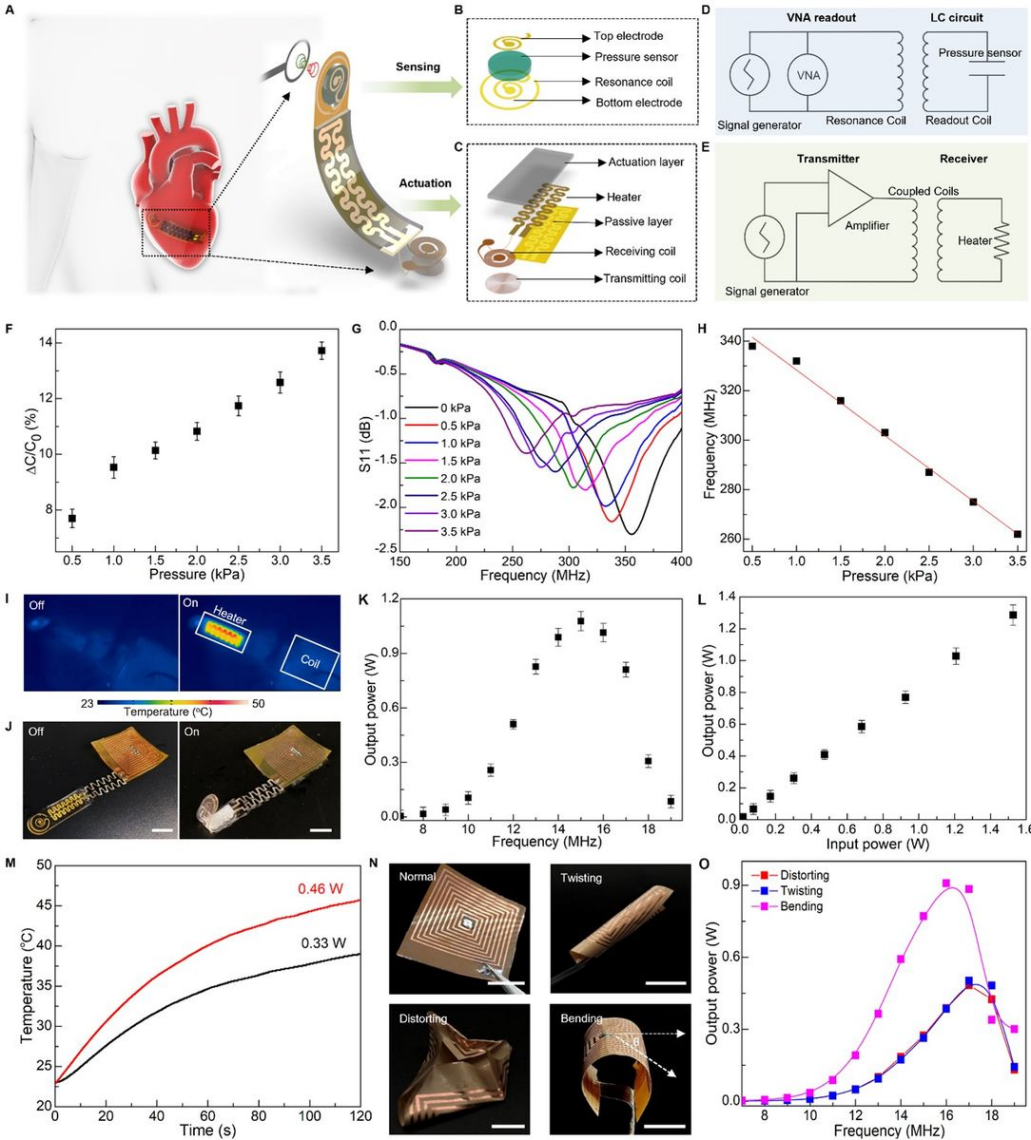
Design and construction of a soft sensory robot for wireless sensing and actuation. **(A)** Schematic illustration of a soft sensory robot constructed with an electrical heater, a polyacrylamide (PAAm)-based pressure sensor, and two inductive coils for transmission sensing signals **(B)** and electrical power **(C)**, respectively. **(B)** Exploded view of the sensing components containing a capacitor formed by two electrodes, a PAAm-hydrogel dielectric layer, and an inductive communication coil made of copper (Cu). **(C)** Exploded view of the actuation components primarily consisting of a PNIPAM actuation hydrogel, a flexible electrical heater, and a radio-frequency (RF) power harvester based on a copper coil. **(D)** Equivalent circuit diagram of the component for wireless pressure sensing. Connecting the pressure-sensing capacitor to an inductor coil forms an LC resonance circuit, where the pressure change on the capacitor leads to the capacitive change and translates to the change of characteristic resonance frequency of the LC circuit, which can be captured by an external probing coil connected to a vector network analyzer (VNA) to realize the wireless sensing function. **(E)** Equivalent circuit diagram of the component for wireless actuation, A transmitting coil connected to an RF power amplifier delivers energy to the receiving coil of the sensory robot, which transmits an electric current to the heater that actuates the robotic motion. **(F)** Measured capacitive change of the PAAm-based pressure sensor in response to applied pressure. **(G)** Measured shift of resonance curves of the PAAm-based pressure sensor in response to applied pressure. **(H)** Change of the LC resonant frequency as a function of applied pressure serving as a signal-transduction scheme for wireless pressure detection. (I) Thermal distribution of the electrical heater and the receiving inductor while wirelessly harvesting energy. The results exhibit minimal heating in the receiving coil with most of the transmitted power consumed by the electrical heater, demonstrating the capability for minimum heat damage to surrounding bio-environments and efficient energy usage. **(J)** Optical images of a soft sensory robot undergoing a wireless actuation to transform from a flat state to a bent state. **(K)** The output power as a function of frequency, showing the optimized frequency is ~15 MHz. **(L)** The output electrical power as a function of the input power. **(M)** The temperature change of the electrical heater overtime under various output powers used for wireless actuation. **(N)** Optical images of the deformed RF coils including bending, twisting and distorting. **(0)** The resonance frequency changes of RF coil under various shape deformations. Here the bending angle *0* is 60°. Scale bars, 5mm.

Figure 4A–C show schematic illustrations of the system design consisting of sensor and actuator components. Our strategy for wireless sensing relies on a passive inductor-capacitor (LC) resonance circuit formed by a planar inductor coil and a parallel plate based on capacitor polyacrylamide (PAAm) hydrogel, as illustrated in Fig. 4B. A change in a biomechanical event (e.g., vessel filling, cardiac contraction/relaxion, and bladder filling/voiding) can lead to a corresponding change in capacitance of the hydrogel-based capacitor, which can be quantified by recording shift in resonance frequency (*f*_*s*_) of the LC circuit according to the equation $f_{s}=1/2\pi \sqrt{LC}$, where *L* and *C* are the inductance and capacitance of the resonance circuit, respectively [74,75].The inductor coil couples to an alternating electromagnetic field through a readout probe, enabling quantitative measurement of the input return loss (S11) using a vector network analyzer (VNA) (Fig. 4D and **Supplementary Note S4**). The proposed PAAm-based sensor with relatively low modulus, intrinsic stretchability, and biocompatibility, can detect the pressure through variation of capacitance between the two electrodes. Figure 4F shows a linear correlation between the measured *ΔC/C*_*0*_ and applied pressure. The slope of the linear fitting curve reports gauge factor (GF) of the sensor reaching ~ 3%/kPa in the clinically relevant range of pressure (0–4 kPa). Figure 4G&H shows the resonant frequency decreases from 340 MHz to 260 MHz in response to the applied pressure decreasing from 0 to 3.5 kPa, and the pressure sensitivity of the sensor is ~ 26.7 kHz/kPa.

Moreover, wireless electromagnetic power transmission is an attractive solution to overcome limitations imposed by implantable systems with discrete batteries, especially for devices operating in enclosed places such as the human body [75–77]. Here, we demonstrate the nature-inspired, sensory robot allows for electrically controlled locomotion in this wireless, battery-free manner (Fig. 4A). Figure 4C shows layout of the wireless actuation design including a PNIPAM actuation hydrogel, a flexible electrical heater, and a radio-frequency (RF) power harvester based on a Cu coil. The RF harvester that is made of a triple-layer structure (**Fig. S27A**&**B**), can be integrated with medical implants (**Fig. S27C** and **Fig. S41D–I**). Magnetic coupling between the transmitting and receiving coil generates electric currents to the heater that thermally stimulates the artificial muscle (Fig. 4E & **Fig. S27D**). As shown in Fig. 4J and **Fig. S28B**&**C**, the robotic finger can undergo a bending motion upon receipt of electromagnetic energy from a power-transfer module. Figure 4I shows an infrared image of the robotic finger highlighting concentrated heat on the electrical heater and minimum heat on the receiving inductor, which collectively ensures a stable power supply and minimum heat damage to surrounding bio-environments in potential usage as implants. Figure 4K shows the output power as a function of frequency. The wireless power transfer system achieves the highest harvested power ~ 1.05 W at a frequency of 15 MHz. Figure 4L indicates the harvested power can be tuned via varying the input power at the optimal frequency 15 MHz, and the power efficiency reaches around 80%, which is sufficient to trigger the heater for raising local temperature to drive motions of the soft robot (Fig. 4M).

The soft robotic system features spatiotemporal locomotion wirelessly controlled by RF signal modulation. To demonstrate the working principle of this method, we fabricate a soft robotic gripper with three arms as an illustrative example. This strategy employs a three-lead LC receiver coil designed based on the frequency-response characteristics of magnetic resonance coupling (**Fig. S29A**&**B**), allowing for the formation of two different configurations with distinct inductive values. These configurations are paired with corresponding capacitors to form LC circuits with different resonant frequencies (**Fig. S29C**). By coupling with different transmission coils operating at distinct transmission frequencies, the coils can selectively deliver power to specific heaters, thereby enabling the activation of individual robotic arms (**Fig. S29D–F**). Furthermore, we conduct a further investigation into the influence of shape deformation, including bending, twisting, and distorting, on the transfer performance of the RF harvester (Fig. 4N). **Fig. S27E** demonstrates that the zeros of the reactance of the receiving circuit remain constant throughout the entire process, indicating minimal disruption to the coil’s resonance frequency [78,79]. Additionally, Fig. 4K and Fig. 4O exhibit a reduction in transmission efficiency resulting from decreased mutual inductance between the transmission and the receiving coils due to shape deformation. For all the deformations shown here, the resonance frequency of the coil remains within an acceptable range for power harvesting, and the power transmitted remains above the minimum level required for the robotic actuation. **Supplementary Note S5** and **Figs. S30–S32** further demonstrate the effect of various design and operational parameters on the power transfer system [80–82]. This yields systematic metrics for designing a well-tuned WPT system, capable of fulfilling the specifications of the targeted application, concurrently decreasing power expenditure, and evading possible risks to living organisms [83,84]. Such wireless robotic systems may serve as a promising solution to safe, real-time monitoring of internal pressure needed for various medical procedures. Notably, **Fig. S27C** shows the circuits for wireless sensing and actuation can be integrated together onto a single e-skin, realizing device miniaturization and efficiency. On the basis of integrated high performance, the confluence of wireless technology and biosensors offers the possibility to detect and manage medical conditions remotely, beyond clinical settings.

### Soft sensory robots interfacing with various internal organs

Soft sensory robots have significant potential for medical-device applications that warrant safe, synergistic interaction with humans [85,86]. The robotic implants developed here can enable minimally invasive implantation. Here, the devices can be stored in a thin catheter and transported safely through small incisions without phase transition until reaching the LCST, in contrast to open surgery where large incisions are required [87,88]. **Fig. S33** provides a schematic illustration of a minimally invasive catheter with a retractable nitinol wire that guides the starfish-structure robot (**Fig. S33A, Fig. S34A, Fig. S34C-F** & **Supplementary Movie S7**) and hand-structure robot (**Fig. S33B, Fig. S34B, Fig. S34G-J** & **Supplementary Movie S7**) to the heart and blood vessel, respectively. As shown in **Fig. S33A**, the delivery involves the following steps: (i) the starfish robot is soft, flexible, resilient and robust, making it easy to be enclosed inside a catheter; (ii) the robot undergoes unfolding motion as it is pushed out of the catheter at the targeted site; (iii) the robot conformally attaches to the epicardial surface due to the inherent softness and adhesiveness of the hydrogel, which accommodates curvilinear surfaces; (iv) the hydrogel layer can be activated by body temperature or joule heating (depending on the LCST of PNIPAAM hydrogel), allowing the starfish-structure robot to form a stress-free interface with the beating heart. Moreover, the device can be further miniaturized to fit within the size constraints of various types of catheters. **Fig. S33B** demonstrates an example of a hand-shape robot approaching the blood vessel via a catheter insertion (**Fig. S33Bi–ii**). Upon stimulation, the robot tightly holds the blood vessel and forms a conformal, stress-free interface (**Fig. S33Biii–iv**). **Figs. S35–S39**, and **Supplementary Movie S8** demonstrate that the skin-inspired soft robots can be designed with a diverse array of configurations, to enable minimally invasive deployment extensive interfacial engagement, and structural reconfiguration to transform between these two stages. Furthermore, in line with pioneering research [89–96], our soft robots feature a profound versatility in geometrical and dimensional design, ensuring a refined adaptability to the specific preferences and requirements of individual cases **(Fig. S40&S41, Table S2**, and **Supplementary Note S7)**. Here, incorporation of acrylamide (AAm) into the PNIPAM hydrogel to form poly(NIPAM-co-acrylamide) (P(NIPAM-AAM)) effectively increases its LCST [64,97–99]. This ensures that the as-prepared robot maintains an unactuated configuration during its passage through the body, while being actuated upon a digitally controlled thermal trigger, thereby providing surgeons with a sufficient time window to perform needed procedures **(Supplementary Note S6** and **Figs. S42–S44)**. Notably, the temperature-dependent hydrogel adhesion could further minimize device migration and delamination due to potential reswelling at a slightly decreased temperature **(Fig. S45&S46**, and **Supplementary Note S8)**. This suggests that our proposed design can be customized and developed to facilitate various types of minimally invasive implantation. *In vivo* tests with artificial organ models demonstrate the versatilities and capabilities of our soft robots for enhanced sensing, stimulation, and drug delivery.

**Figure 5:**
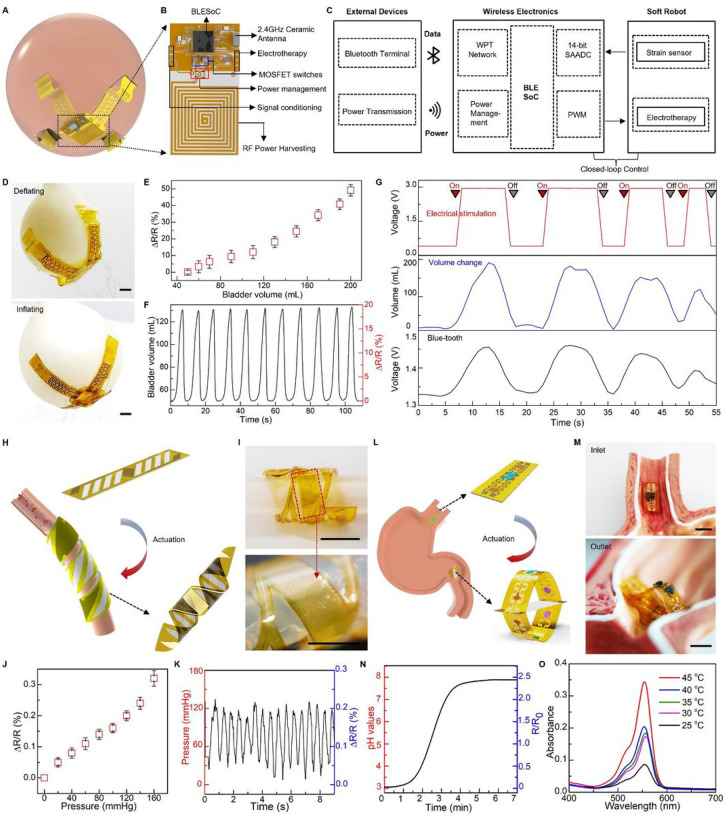
Soft sensory robots interfacing with various internal organs. **(A)** Schematic illustration of a fully implantable, soft robotic gripper gently holding a bladder for precise measurement of bladder volume and providing electrical stimulation in a wireless closed-loop control fashion. **(B)** The control platform consists of a wireless power harvesting network, a full bridge amplifier, a voltage regulator, a signal conditioning circuit for strain sensors, a Bluetooth System-on-Chip, and a MOSFET switch for amplification of electrical stimulation. **(C)** System block diagram of the wireless closed-loop controlled bladder electrical stimulation module. **Fig. S47A** illustrates the structural design of the soft robotic gripper composed of bilayer actuators and buckled strain sensors. **(D)** Demonstration of the soft robotic gripper deployed onto an artificial bladder based on a balloon undergoing repetitive transformations from a deflating state to an inflating state. Here, the 3D buckled strain sensor attached onto the balloon enables real-time detection of bladder volume as shown in **Fig. S47B. (E)** Measured resistive characteristics of the buckled strain sensor on the artificial bladder as a function of the bladder volume. **(F)** Representative test of the 3D buckling strain sensor in real-time monitoring of volumetric change of the artificial bladder during cyclic movements of filling and emptying. **(G)** Programmed electrical stimulation (top) and measured volume of an artificial bladder based on a balloon (middle and bottom). The experimental demonstration is conducted using the following parameters: volume threshold of ~100 mL, electrical stimulation amplitude of 3 V. **(H)** Schematic illustration of a soft robotic cuff integrated with a strain sensor enclosing around a blood vessel for monitoring blood pressure. **Fig. S50A** shows the layout of the soft robotic cuff. (I) Top: Optical image of a soft robotic cuff wrapping around an artificial vessel made by a rubbery tube through which water is pumped to stimulate blood circulation. Bottom: Enlarged view of the robotic cuff highlighting the integrated strain sensor made of a serpentine Au/PI resistor for measuring blood pressure. **(J)** Measured resistive change of the strain sensor at various simulated blood pressures. **(K)** Representative measurement of fluidic pressure of the artificial artery system using the soft robotic cuff. **(L)** Schematic illustration of a soft ingestible robot designed for continuous monitoring of pH and extended drug delivery inside the stomach. The robot consists of the actuation layer (bilayers of PNIPAM and functional nanocomposites), drug-releasing layer (PLGA/rhodamine-B patch composite), and the pH sensing layer (PEDOT:PSS/PVA hydrogel) as shown in **Fig. S51A (M)** Optical images showing the soft ingestible robot entering (top), expanding and blocking (bottom) in the stomach. This engineering design enables the drug patch and pH sensor extensively retained inside stomach to enhance the efficiency and precision of drug release and pH monitoring. **(N)** Electrical response of PEDOT:PSS/PVA hydrogel to pH change ranging from 3 to 7 over time. (0) Here, rhodamine-B is used as a model substance that is embedded into the poly lactic-co-glycolic acid (PLGA) matrix to form a drug delivery patch concealed inside the robot. The UV-vis absorbance spectrum is used to measure the concentration of the rhodamine-B released from the robot in 1 h under various temperatures which dictate the robotic motion and further affect the exposure area of the drug patch to the gastric fluid. Scale bars, 5mm.

Urinary bladder dysfunction is one of the emerging issues in an aging society, which not only leads to loss of voluntary control over the bladder muscles, but also cuts off sensorial feedback to central nervous system [100], In most cases of bladder dysfunction, the patients are not able to sense the fullness of bladder with urination, making it a challenge to time the action for voiding treatment (e.g., electrical stimulation) [101]. Therefore, realizing the voiding treatment in a timely manner requires a monitoring system that measures the bladder status continuously. Here, we develop a soft robotic gripper for both real-time assessment of bladder volume and voiding treatment in a wireless closed-loop control fashion. The as-prepared robotic system, illustrated in Figs. 5A–5C& **Fig. S47A** consists of a flexible hydrogel-based actuator, a 3D buckled strain sensor, an electrical stimulator, and a control module. The actuator includes a passive layer made of a patterned Au/PI bilayer as an electrical heater, and an active layer of PNIPAM hydrogel. Upon an electrical trigger delivered via an inductive coil, the robotic gripper can bend and wrap around the bladder conformally and gently (Fig. 5A) to ensure precise measurement of bladder volume and minimized stress at the interface. The strain sensor integrated into the robot is capable of detecting bladder volume continuously. Here, the strain sensor includes an elastic PAAm hydrogel film and a serpentine Au/PI resistor to form a buckled 3D structure for enhanced sensitivity. The fabrication steps for the buckled sensor appear in Materials and Methods. We use a balloon model to mimic the natural bladder behavior of filling and emptying to validate performance of the integrated soft robotic gripper. **Fig. S47B** shows the sensor conformally attaches onto the balloon surface with the biocompatible adhesiveness of PAAm hydrogel [102]. The softness of PAAm hydrogel in the robot achieves minimal strain disruption to the bladder during the device operation. Injecting and extracting water into the balloon with a syringe pump realize the behavior of bladder filling and emptying (Fig. 5D). The change of sensor resistance exhibits a strong linear correlation with bladder volume during its expansion and shrinkage (Fig. 5E, and **Fig. S47C&D)**, thus serving as an indicator in bladder-volume control. Figure 5F demonstrates the system repeatability in real-time monitoring during multiple cycles of bladder filling and emptying.

The readout of the bladder volume is achieved through a voltage divider circuit (VDC) consisting of a reference resistor connected in series with the as-fabricated strain sensor. This configuration has minimal impact on the wireless power transmission between coils **(Fig. S48)**. To achieve a closed-loop control of electrical stimulation in the treatment of a dysfunction bladder system, we further program the Bluetooth-Low-Energy (BLE) System-on-Chip (SoC) (BLE SoC) to enable a pulse-width modulation (PWM) instance. The instance, together with on-board power amplification and filtering circuits, facilitates the application of programmable electrical stimulation in response to an increased strain in the bladder. This enables on-demand electrotherapy and closed-loop control of the robotic implant, as illustrated in Fig. 5B and Fig. 5C. A demonstration of the control scheme can be found in **Fig. S49** and Fig. 5G. When the balloon reaches a certain volume threshold, the control system triggers a command for electrical stimulation.

The on-demand motion provided by the soft robot facilitates device implantation and ensures benign and stable contact with targeted tissue or organs [10,103,104]. Here we develop a soft robotic cuff that can enclose around a blood vessel upon thermal stimulation (Fig. 5H), to enable real-time measurement of blood pressure. As shown in **Fig. S50A** the soft robotic cuff consists of a muscle layer based on a PNIPAM and an e-skin layer embedded with a strain sensor based on a serpentine Au ribbon. Notably, the e-skin layer uses a pattern of parallel strips in the PI film, that forms a 45° angle with the longitudinal direction of the device, which, by coupling with the muscle layers, facilitates formation of a helix structure. An in vitro model of artery that uses a rubbery tube with a pulsatile flow of water to create a simulated pulsation pattern validates performance of the soft robotic cuff **(Fig. S50B&C** and Fig. 5I). The helix formation of the robotic cuff provides a gentle and stable coupling with the artificial artery, of which the measured signal (resistive change) exhibits a linear relation with the inner fluid pressure (Fig. 5J). Figure 5K demonstrates the capability in continuous measurement that captures patterns of simulated pulsation, further confirming its utility in measuring blood pressure.

Furthermore, such nature-inspired designs of soft sensory robots can also configure into an ingestible platform. Here, we show that a soft ingestible robot is capable of prolonged residence in the stomach for pH monitoring and drug delivery. **Fig. S51A** depicts the proposed structure of the ingestible robot based on a slab-shaped muscle layer sandwiched by two e-skin layers consisting of a drug delivery module and a pH sensing module (Fig. 5L, more details appear in the Materials and Methods.). The miniaturized size of the robot facilitates swallowing and transport through the esophagus to stomach (Fig. 5L). Once in the stomach, the robot self-expands that prevents passage through the pylorus for an extended duration in the gastric environment (Fig. 5M and **Fig. S51B)**. The soft pH sensors based on PEDOT:PSS/poly(vinyl alcohol) (PVA) embedded onto the e-skin of the robot provide continuous measurements of gastric pH **(Fig. S51C** and Fig. 5N). The pH sensor exhibits a linear relationship between the resistive change and pH of the fluidic environment within the range of acidic (pH ~ 3) to basic (pH ~ 8) **(Fig. S51E)**. To realize prolonged drug release, the integrated drug patches use poly(lactic-co-glycolic acid) (PLGA) as a matrix to load drugs and the robotic motion as a trigger mechanism that exposes the drug-loaded patches to stomach fluid for initiating drug release. As a demonstration, we use a biocompatible dye, rhodamine-B (RB), as a model drug (5 mg of RB per 0.5g PLGA). Figure 5O and **Fig. S51H** show the UV-vis absorption spectra and the corresponding dosages, respectively, of the RB-loaded drug patch, immersed in PBS solution (at pH ~ 5) for 1 h under various temperatures ranging from 25 °C to 45 °C, **(Fig. S51 F&G** show the calibration curve based on the measured UV-vis absorption spectra.). Such ingestible soft robot highlights a synergistic combination between sensing function and robotic motion to realize on-demand, prolonged control of drug delivery.

### A soft robotic thera-gripper for epicardial sensing and pacing

**Figure 6:**
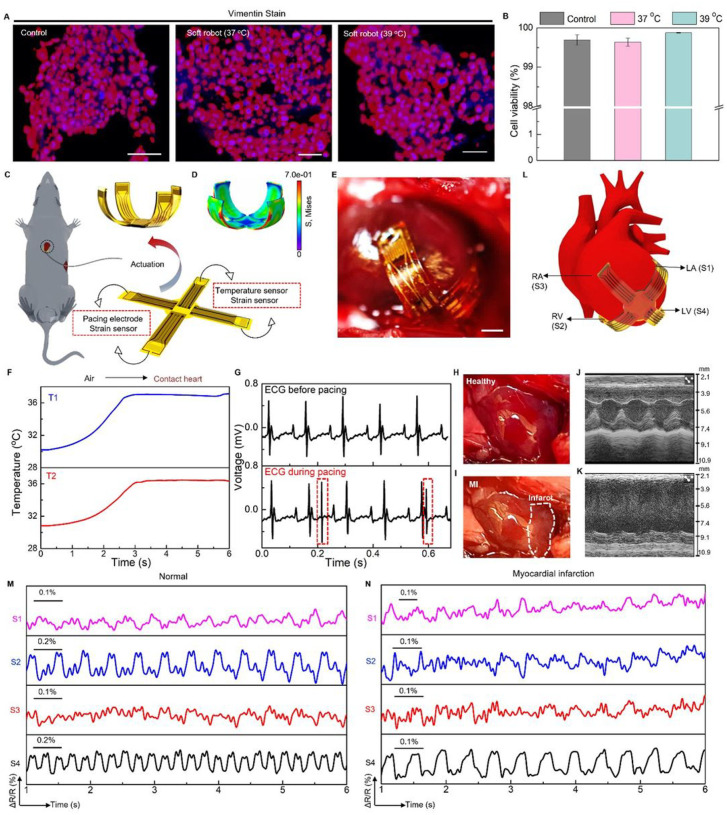
*in vivo* validation of a soft robotic thera-gripper for epicardial sensing and pacing. **(A)** Confocal microscope images of 3T3-J-2 cells before (control) and after exposure to as-prepared soft robots integrated with an e-skin layer and a PNIPAM hydrogel-muscle layer, incubated at 37 °C and 39 °C for 48h. Scale bars, 50 μm. **(B)** Comparative cell viability before and after soft robot’s exposure, indicating that 3T3-J-2 cells exposed with the as-prepared soft robot high and cultured at the elevated temperature (39 °C) have no decreased viability. Scale bars, 50 μm. **(C)** Schematic illustration showing the thera-gripper features minimally invasive insertion at the resting state and wraps onto the surface of a beating heart at the actuation state. The thera-gripper contains four strain sensors made of serpentine Au/PI resistors, two pacing electrodes based on Au, and two temperature sensors made of thermal resistors. An exploded schematic view of the soft robotic thera-gripper is shown in **Fig. S52A. (D)** Finite element modeling of the actuation state. The colors in the legend indicate the magnitude of the von Mises stress. **(E)** Image of a soft robotic thera-gripper grasping on the epicardial surface of a living mouse heart. Scale bar, 5mm. **(F)** Temperature measurements from the thera-gripper during its deployment onto the mouse heart. **(G)** The surface ECG trace during electrical stimulation using a pair of Au pacing electrodes. **Fig. S52D** shows the representative voltage traces of the ECG under electrical stimulation with different voltages, offering great potential for selectively and locally pacing the cardiac cells to restore normal heart function. (H&l) Optical images of a healthy heart (H) and heart two weeks after myocardial infarction (Ml) **(I)**. The Ml area is shown by the white dashed circle in **(I). Fig. S63B** shows the post-MI ECG signals with hyperacute T waves. **(J&K)** M-mode echocardiographic images from a healthy **(J)** and post-MI heart **(K). (L)** Schematic illustration showing the thera-gripper position on a mouse heart, where the strain sensors (labeled as SI, S2, S3, and S4) are located onto different heart chambers for locally monitoring of dysfunctional tissue. **(M&N)** Representative measurements of local cardiac contractions before **(M)** and after myocardial infarction **(N)** using a soft robotic thera-gripper wrapping onto a living mouse heart.

Cardiac implants that can monitor and regulate heart rhythms are critical for patients with severe cardiac diseases [105,106], Despite the broad implementation of cardiac implants or pacemakers in clinical settings, however, existing devices are usually rigid, undeformable, and lack structural reconfigurability to adapt to dynamic motions of beating heart, which precludes optimum performance chronically and safely [107]. Here, we report a soft robotic thera-gripper that can gently envelop a heart to perform spatiotemporal monitoring of electrophysiological activity, temperature, and strain, and provide therapeutic capabilities (e.g., electrical pacing). The soft robotic thera-gripper contains four multi-functional arms, emulating a starfish, to ensure effective latching onto the epicardial surface of which the soft mechanics ensures negligible disruption to cardiac dynamics. **Fig. S52A** presents an exploded view of the robotic thera-gripper, which contains actuators based on PINPAM actuation hydrogel, two temperature sensors made of thermal resistors, two pacing electrodes made of Au, and four strain sensors made of serpentine Au/PI resistors (The fabrication approach appears in Materials and Methods, and **Fig. S52B.)**. Figure 6C shows the thera-gripper at its resting state features minimally invasive insertion with a medical catheter, and forms a bowl shape at the actuation state, via a slightly raised temperature, to gently hold a beating heart for a safe, stable interface (Fig. 6C). Figure 6D shows the corresponding FEA result of an actuated soft robotic thera-gripper. Moreover, we examined the biocompatibility of the soft robots *in vitro* and *in vivo*. Here, mouse 3T3-J-2 cells exposed to complete soft robotic devices with constituent materials including pure PNIPAM hydrogel and functional nanocomposites (e.g., AgNWs, RGO, PEDOT:PSS & MXene), remain robust healthy and maintain stable viability (Fig. 6A, Fig. 6B, **Fig. S53** & **Fig. S54)** [108,109]. Additionally, histological analysis reveals that the soft robots implanted inside chest chamber of mice induce no observable inflammation or other adverse effects to surrounding tissues, indicating good biocompatibility for long-term operation **(Fig. S55** & **Fig. S56)**. The incorporated temperature sensors provide feedback information on device temperature that govern the structural transformation, upon contact with cardiac tissues (Fig. 6F). The pacing electrodes integrated into our thera-gripper can generate electrical impulses to regulate cardiac pacing (Fig. 6G and **Fig. S52C&D). Fig. S57** shows measured ECG traces, highlighting effective transmission of electrical impulses (voltage ranges from 500 mV to 2 V with 1 ms width at 2.65 Hz) onto cardiac tissues. The e-skin layer consists of microelectrodes for capturing electrical activity of heart, which serves as essential guidance in operating electrical stimulation/pacing **(Fig. S58). Figs. S59–S61** showcase simultaneous sensing and stimulation from a robotic thera-gripper on a beating heart with an *in vivo* mouse model, demonstrating its capability in closed-loop operation of cardiac pacing.

Cardiovascular disease is one of the leading causes of death worldwide affecting more than 17.9 million people per year [110]. Real-time and continuous monitoring of myocardial functions (e.g., contractility) is desired for patients with high risks of cardiac arrest, which not only ensures proactive treatment prior to occurrence of adverse events, but also provides comprehensive evaluations of therapeutic effects during postoperative care [111–113]. A soft thera-gripper, integrated with strain sensors distributed on its arms, can provide continuous, spatially resolved quantification of myocardial strains, holding great potential in precision treatment for cardiac diseases. Here, a chronic model of myocardial infarction (Ml) with permanent ligation of the left coronary artery (LCA) of a mouse enables assessment of the sensing capability of a thera-gripper post to its robotic motions to establish optimized sensing interfaces (details appear in Materials and Methods.). Figure 6H&I show the infarcted area that turns into pale myocardium. Echocardiographic strain imaging provides continuous quantification of regional myocardial function. **Fig. S62B&C** show the short-axis views in echocardiographic imaging (B-mode) from a normal and post-Ml left ventricle (LV) of heart, respectively. The M-mode images, as shown in Fig. 6J&K, display the fraction shortening (FS) decreases from 75–15% upon occurrence of Ml, indicating the heart exhibiting moderate dysfunction accompanied by regional contractility loss **(Supplementary NoteS9** and **Fig. S62D&E)** [1 14,115]. Although echocardiography is an invaluable tool allowing for reliable diagnostic information necessary for the clinical decision, it often lacks convenient accessibility and usually takes more than 30 minutes to capture the movement and function of the heart muscle and heart valves [116]. Figure 6L and **Fig. S63C** show that the multi-strain sensors (labeled as S1, S2, S3, and S4) of the implanted thera-gripper distributed across four chambers of the heart, respectively, highlighting the key advantage of our thera-gripper with real-time strain sensing over traditional imaging tools in recording local contractions continuously and simultaneously. **Fig**. S63A shows the response of a representative strain sensor, revealing mechanical rhythms of the cardiac cycles, consistent with ECG recordings. Ligation of coronary artery leads to myocardial infarction that causes ST-segment elevation, as shown in **Fig. S63B**. Figure 6M&N show the contractility patterns of the right and left atria (RA and LA), and the right and left ventricles (RV and LV) under normal and Ml conditions, respectively, measured by an implanted thera-gripper where the output features of the sensors are determined by their experienced strain that significantly correlates to their positions on epicardial surface. The S4 (LV) displays the largest amplitude due to a maximum experienced strain, indicating the intrinsic highest myocardium strength in the LV chamber. Figure 6I reveals the area of infarction two weeks after the Ml surgery. The results in Fig. 6N demonstrate that the LV outflow obstruction changes patterns of ventricular contraction. The sensors S2 (RV) and S4 (LV) experience a reduced strain change due to the consequent loss of contractile myocardium, which, correspondingly, reduces force of myocardial contractility and decreases heart rate. Collectively, such nature-inspired design establishes a foundation for implantable robots to harness on-demand motion for structural adaptation inside body and sensory functions for real-time optimization of therapeutic outcomes.

## Discussion

In this study, we report concepts and device designs to achieve untethered soft robots that highly emulate biological systems and seamlessly integrate actuators, sensors, and stimulators to enable structural adaption and reconfigurable interfaces that minimize tissue damage *in vivo*, enhance mechanical match at biotic-abiotic interface, increase biocompatibility, and improve multimodal functionality with spatiotemporal precision. Our soft robots primarily consist of an e-skin layer made of multi-modal nanocomposites that mimic receptors in biological skin to perceive various external stimuli, and an artificial-muscle layer made of thermally responsive PNIPAM hydrogel to generate adaptive motion. We employ an *in situ* solution-based approach to fabricate the flexible multi-modal e-skin. This facile method represents a versatile platform, for a broad range of functional materials (e.g., AgNWs, RGO, and PEDOT:PSS) to be incorporated into a polymeric matrix (e.g., PDMS and PI) to form various types of sensors (e.g., temperature, pressure, and strain) with high spatiotemporal resolution. Such biomimicry design of soft robots offers versatility in mechanical motions including bending, twisting, and expanding as well as diversity in structural deformation, including configurations resembling starfish, fishbone, chiral seedpod, and others. On-demand actuation triggered by electrothermal stimulation from electrical heaters embedded in the e-skin allows precise, independent control of regional parts of the soft body. Furthermore, integration with wireless modules enables the robots to be controlled and communicated without tethering, even when implanted inside body. To demonstrate the broad utility, we develop soft robots that are tailored for specific application scenarios. Specifically, we fabricate a soft robotic gripper that can wrap around a bladder to enable coordinated, closed-loop operation of bladder-volume evaluation and electrical stimulation to treat an overactive bladder, a robotic cuff that can twist around a blood vessel for measuring blood pressure, and an ingestible robot that can reside in a stomach for prolonged pH sensing and drug delivery. *In vivo* studies with a mouse model demonstrate capabilities of a soft robotic thera-gripper in gently enveloping a beating heart, spatiotemporal assessment of electrophysiological activity, quantification of cardiac contractility, and supplying electrical stimulation for pace regulation. These demonstrations showcase the potential applications of such soft robots as next-generation biomedical implants with structural intelligence and multi-functionalities. Future advancements could further enhance the synergistic interaction between soft implantable robots and biological tissues, to achieve long-term biocompatibility and stability in dynamic physiological environments for improving treatment of chronic diseases.

## Materials and methods

### Materials:

N-isopropylacrylamide (NIPAM, 98%) was purchased from TCI. Poly (vinyl alcohol) (PVA, 99+% hydrolyzed), poly(D,L-lactide-co-glycolide) (Mw 50,000–75,0000), acrylamide (AAm, 99%), N, N’-Methylenebisacrylamide (BIS, 99%), N,N,N’,N’-Tetramethyl-ethylenediamine (TMEDA, 99%), and ammonium persulfate (APS, >98%) were purchased from Sigma-Aldrich. Rhodamine B and silver nitrate (AgN0_3_ 99.9% ) was purchased from Themo Scientific. Ethylene glycol (EG, >99%) was purchased from BDH Chemicals. Polyvinylpyrrolidone (M_w_ ≈55000, PVP) and poly(3,4-ethylenedioxythiophene)-poly(styrenesulfonate) (PEDOT:PSS, 3.0–4.0%) were purchased from Sigma-Aldrich. Copper chloride (CuCl_2_) was purchased from Ward’s Science. The graphite powder was purchased from Spectrum Chemical Manufacturing Corp. The sodium nitrate (NaN0_3_, >99%), hydrogen peroxide (H_2_0_2_,30% w/w), the potassium permanganate (KMn0_4_, >99%), hydrochloric acid (HCI, 36.5–38%) and sulfuric acid (H_2_S0_4_,95%–98%) were purchased from BDH chemicals. Ethylenediamine (EDA, 99%) was purchased from Alfa Aesar.

### Materials characterization

The SEM images were taken by the field emission scanning electron microscope (Hitachi S-4700 with EDS). The XPS spectra were obtained by Kratos Axis Supra x-ray photoelectron spectrometer, allowing to determine the elemental composition of the top ~ 10 nm of the sample surface. The XRD patterns of functional nanocomposites were obtained using the Rigaku SmartLab theta-theta diffractometer. The FTIR spectra were recorded using Hyperion 1000 with Tensor 27 spectrometer. The thermal images were taken with an infrared (IR) camera (FLIR ETS320). The performance of the drug-release patch was characterized with a Ultraviolet-visible (UV-vis) spectrometer (VWR UV-1600PC).

### Multi-modal sensory soft robots with nature-inspired designs

#### The fabrication of thermo-responsive hydrogel

Poly(N-isopropylacrylamide) (PNIPAM) was synthesized based on precipitation polymerization. In a typical example, 500 μL NIPAM as monomer (25 wt%), 50 μL BIS as crosslinker (0.5 wt%), 140 μL APS (1 wt%)/200 μL TEMED (2 wt%) as initiator were mixed to form the precursor solution. After 30 mins, the precursors can polymerize, *in situ*, to form the thermos-responsive PNIPAM hydrogel.

#### The fabrication of AgNWs

AgNWs were synthesized based on a modified polyol method. 100 mL of EG containing NaCl (~ 0.05 mM), PVP (~ 189 mM), AgN0_3_ (~ 0.0014 mM), and CuCI_2_ (~ 0.017 mM) were added to a round-bottom flask and heated at 185 °C for 1 h in an oil bath. Then, 30 mL AgN0_3_ EG solution (~ 0.12 M) was added dropwise with vigorous stirring. After the reaction was completed, the flask was cooled to room temperature. The AgNW suspension in the EG was diluted with 30 mL water and sedimented for 14h. The supernatant was decanted, the water was added to reach 160 mL in total volume of the solution. The suspension was sedimented for 14h and decanted. Then, 50 mL water was added to this mixture, and 100 mL acetone was slowly added with gentle mixing. After centrifugation, the as-formed AgNW pellets was fully resuspended in a 20 mL PVP aqueous solution (~ 0.5 wt%). The cleaning process was repeated 4 times. Finally, the AgNW pellets were stored in water for later use.

#### The fabrication of reduced graphene oxide

The graphene oxide (GO) was synthesized based on a modified Hummers method. In general, 5 g of graphite powder and 2.5 g of NaN0_3_ were added into 120 mL sulfuric acid in an ice bath under stirring for 2h. Subsequently, 15 g of KMn0_4_ was slowly added into the solution under a temperature < 20 °C. After 1 h, the reaction temperature was raised to 35 °C for overnight reaction. 150 mL H_2_0 was added to the mixture for 12 h reaction under 98 °C. Then, 500 mL of H_2_0 and 20 mL of H_2_0_2_ were added to the mixed solution. Finally, the solution was washed with HCI (1M) and H_2_0 until the pH was natural. The EDA was used as the reducing agent to reduce the graphene oxide.

##### The fabrication of a soft robot inspired by a starfish:

The fabrication process of the multi-modal functional electronic-skin (e-skin) is shown in **Fig. S2A**. A polyimide (PI) substrate (thickness ~ 10 μm) was patterned on a pre-cleaned and plasma-pretreated glass silde using laser-cutter (six rectangular, each is 15mm × 6mm). The AgNWs solution (50 wt%), the RGO suspension (5 wt%), and PEDOT:PSS solution were dropcast onto the patterned glass slide and heated at 50 °C for drying. After the solution was dried, the structured functional materials were fabricated with laser cutting (SFX-50GS). Then, a thin layer of liquid PI was spin-coated onto the patterned thin film of functional nanomaterials and cured at 150 °C for 1h. Finally, the resultant film was cutted out with a laser beam and peeled off from the glass slide. The as-formed multi-modal e-skin was encapsulated with a layer of parylene (thickness ~ 2 μm) and bonded onto PNIPAM hydrogels via the adhesive glue (3M Vetbond 1469c) (Fig. 2C) to generate the multi-modal sensory soft robot with a nature-inspired starfish design.

### The fabrication of a soft robot inspired by chiral seedpods

The gold nanomembrane (Au, thickness ~ 200 nm) and adhesive chromium layer (Cr, thickness ~ 10 nm) were deposited by the magnetron sputtering on the PI film (thickness ~ 10 μm). The patterns for Au electronics and PI substrate shown in **Fig. S8A** were formed using the laser cutting machine. Then the e-skin made of patterned Au/PI film and encapsulated by a parylene layer (thickness ~ 2 μm), is attached to the PNIPAM hydrogel (15mm × 6mm) using the bioadhesive layer.

#### The fabrication of a soft robotic pill:

Firstly, the PEDOT :PSS solution was dropcast onto a pre-treated glass slide spacer, dried under 50 °C and patterned using a laser-cutter. Secondly, the PEDOT:PSS/PI nanocomposite thin film was formed by spin-coating liquid PI onto the patterned PEDOT:PSS, and fully curing at 150 °C for 1 h. Next, the e-skin layer received a protective coating of parylene, ~ 2 μm in thickness. Finally, two pieces of PEDOT:PSS/PI nanocomposite film were adhered to a piece of PNIPAM hydrogel at the edges **(Fig. S10A)**.

### Static finite element analysis for various soft robots

3D finite element analyses (FEA) in commercial software ABAQUS were utilized to predict the shape transformation process of soft robots with different patterns and dimensions. The elastic modulus (*E*) and poison’s ratio (*u*)) used in the simulations were *E*_*PNIPAM*_=90 KPa, *u*_*PNIPAM*_ = 0.30 for PNIPAM hydrogel, and *E*_*PI*_=2.5 GPa, *u*_*PI*_ = 0.34 for PI.

### Wireless sensing and actuation of soft sensory robot

#### The fabrication of polyacrylamide (PAAm) hydrogel pressure sensor

In polyacrylamide (PAAm) hydrogel synthesis, 500 μL AAm as monomer (25 wt%), 50 μL BIS as crosslinker (0.5 wt%), 140 μL APS (1 wt%)/200 μL TEMED (2 wt%) as initiator were mixed to form the precursor solution. After 10 mins, the precursor can polymerize, *in situ*, to form the PAAm hydrogel. Then a parallel-plate capacitor-based pressure sensor was formed through sandwiching PAAm hydrogel between two electrodes made of Au/PI bilayer (Fig. 4B).

#### The fabrication of the soft sensory robot

Laser cutting of the Au/PI bilayer formed the electrical heater that was connected to a Cu receiver coil (Fig. 4C). This assembly was then covered with a thin layer of parylene (thickness ~ 2 μm). Following this, the PNIPAM muscle was bonded to the electrical heater to form the bilayer structure. Finally, the actuation component was integrated with the PAAm-based pressure sensor (Fig. 4A). Here, the circuits for sensing and actuating can be integrated together onto a single piece of Au/PI bilayer film **(Fig. S27C)**.

#### The characterization of wireless power transmitting and pressure sensing

The detailed power transfer performance and pressure sensing data acquisition are explained in **Supplementary Note S3**.

#### Wireless control of locomotion

We implement a three-lead LC receiving network based on the frequency response characteristics of magnetic resonance coupling **(Fig. S29A)**. In this system, two leads are connected to the full length of the coil, while a third electrode is connected to the middle of the coil loops, resulting in the formation of a smaller inductor with the common electrode **(Fig. S29B)**. The two coil loops with different inductive values are paired with different capacitors (Full length ~ 200 pF; Half length ~ 47 pF) to form LC circuits with different resonant frequencies yet similar small quality factors **(Fig. S29C)**. Each soft robotic arm is connected to an individual pair of leads to harvest RF power transmitted at different frequencies.

### LCST tunability of PNIPAM-based hydrogel

#### The synthesis of poly(NIPAM-co-acrylamide) (P(NIPAM-AAm)) hydrogel

The synthesis of the PNIPAM-co-PAAm was conducted through a free radical polymerization method. In a typical procedure, a mixture of 450 μL NIPAM monomer (25 wt%) and 50 μL AAm monomer (at concentrations of 5 wt%, 10 wt%, 15 wt%, 20 wt% and 25 wt%) was prepared. Additionally, 50 μL of BIS crosslinker (0.5 wt%), 140 μL of APS initiator (1 wt%) and 200 μL of TEMED (2 wt%) were added to form the precursor solution. After 30 mins, the precursors can polymerize to form the thermos-responsive P(NIPAM-AAm) hydrogel.

#### Measurement of bending angle of soft robots based on PNIPAM-co-PAAm hydrogel.

The bending angles were measured as a function of time under different electrical powers in both a simulated *in vivo* environment (37 °C, PBS solution) and an in vitro condition (room temperature, PBS solution). The measurements were performed for three different levels of AAm incorporation: 0 wt%, 5 wt%, and 10 wt%.

### A soft robotic gripper for bladder control

#### The fabrication of a robotic gripper

The process began with the fabrication of e-skin layer via laser cutting Au/PI bilayer film and applying a parylene coating. The e-skin layer includes electrical heaters, electrical stimulators, and serpentine Au resistors **(Fig. S47A)**. Then, the PNIPAM hydrogel was adhered onto the electrical heater, and the Au/PI resistor was anchored onto one piece of PAAm hydrogel at the edges to form a strain sensor with a buckled structure **(Fig. S47B)**.

#### Measurement of biomimetic bladder volume

A balloon model was used to mimic the natural bladder to evaluate the actuation and sensing performance of the soft gripper. After thermal stimulation, the soft gripper can wrap around the balloon and the strain sensor can attach onto the balloon surface via the PAAm hydrogel. A syringe pump was used to inject and extract water into the balloon to imitate the filling and emptying behavior of natural bladder. The PowerLab (Model 16/35, AD Instruments) allowed the recording of output signals.

#### Wireless sensing of resistive strain sensor and close-looped control of bladder electrical therapy robot

A power harvesting and signal conditioning circuit was fabricated and soldered using the method of soft PCB fabrications **(Supplementary Note S4)**. We developed an alternative approach for powering and signal conditioning. This strategy enables the accurate readout of the resistive strain sensor without significantly affecting the wireless power transmission between coils. Specifically, we fabricate a full-bridge rectifier with surface-mount diodes, enabling the conversion of the alternative current (AC) obtained via the receiver coil into direct current (DC). Subsequently, the rectified DC is fed into a 3.3V low-dropout (LDO) regulator. The output from this regulator serves as the power source for both a Bluetooth-Low-Energy (BLE) System-on-Chip (SoC) and a voltage divider circuit. The voltage divider circuit consists of a reference resistor connected in series with the as-fabricated resistive strain sensor. The voltage divider circuit consists of a reference resistor connected in series with the as-fabricated resistive strain sensor. The voltage across the resistive strain sensor through the voltage divider circuit was sampled by a 14-bit on-chip Successive Approximation Analog to Digital Converter (SAADC). A custom BLE service transmits the sampling value to host terminals acting as BLE clients and parsers to allow wireless readout of strain values. For achieving a closed-loop control of electrical stimulation in the treatment of dysfunctional bladder system, we further programmed the BLE SoC by enabling a pulse-width modulation (PWM) instance. The instance, combined with on-board power amplification and filtering system, enables the programmed electrical stimulation corresponding to detected bladder strain variations.

### A robotic cuff for vascular system

#### The fabrication of a robotic cuff

Firstly, we used laser cutter to fabricate the e-skin layer with a pattern of parallel strips that exhibit a 45° angle with the longitudinal direction of the device. The e-skin layer contains a serpentine Au ribbon as a strain sensor **(Fig. S50A)**. Subsequently, this layer was coated with a parylene film ~ 2 μm in thickness. Finally, the PNIPAM hydrogel-muscle layer was bonded to the e-skin layer.

#### Measurement of biomimetic blood pressure

A rubbery tube was used to mimic the artery system to evaluate the performance of the soft robotic cuff. The soft robotic cuff can twist around the artificial artery upon thermal stimulation. A pulsatile flow of water was created to simulate the pulsation pattern of the artery system. The measured signals of the strain sensor were recorded with the PowerLab.

### An ingestible robot for digestive system

#### The fabrication of a drug-releasing patch

In brief, 5 mg poly(lactic-co-glycolic acid) (~ PLGA) was dissolved in 5.1 g acetone containing 5 mg Rhodamine B (~ RhB). Then the mixture was poured into a poly(dimethylsiloxane) (~ PDMS) spacer, and acetone was removed from the mixture under vacuum condition. Finally, the as-formed film of PLGA/RhB was cut into small pieces of drug-release patch with a diameter ~ 2 mm.

#### The fabrication of PEDOT.PSS/PVA hydrogel sensor.

For hybrid hydrogel fabrication, first a 10 wt% PVA solution was made by dissolving PVA powder in water. Then 5 wt% PEDOT:PSS aqueous dispersion was added into the PVA solution followed by slow mixing for 24 h. The prepared PEDOT:PSS/PVA solution was poured into glass spacer, followed by freezing at −20 °C for 8 h and thawing at 25 °C for 3 h for three times.

#### The fabrication of a soft ingestible robot.

The process began with the formation of e-skin layer. The circuits for sensing were fabricated on Au/PI bilayer film with a laser cutter, while the remaining parts received a parylene coating. The PEDOT:PSS/PVA hydrogel pH sensors were adhered onto the side of Au/PI film with conductive electrodes, while the drug-release patches were integrated onto the other side. Then, two pieces of e-skin layer were adhered to a piece of PNIPAM hydrogel at the edges, as shown in **Fig. S51A**.

### Quantification of RhB release from the drug patch

The drug-release patches of PLGA/RhB mixture were immersed into phosphate-buffered saline (~ PBS) solutions (pH ~ 5) for 1 h under different temperatures ranging from 25 to 45 °C. The UV-vis absorbance spectrometer was used to analyze the amount of the RhB released from the drug-patch.

#### Measurement of pH sensitivity.

The PEDOT:PSS hybrid hydrogels were submerged into PBS solutions with different pH values ranging from 3 to 8. In 5 mins, hydrogels were removed from PBS solutions and their resistance was measured with PowerLab. The effect of pH on the electrical properties of the PEDOT:PSS/PVA hydrogel is attributed to the ionic interaction between PEDOT and PSS polymer chains. Under acidic conditions, PEDOT chains are uniformly distributed along the PSS polymer chains, ensuring the formation of continuous electrical connections between PEDOT segments. While pH shifts from acidic to more alkaline, the homogenous distribution of PEDOT along the PSS polymer chains is interrupted by negatively charged hydroxy groups, and buried inside the insulating PSS phase, as illustrated in **Fig. S51D**.

### Cell morphology analysis, cell viability test and histological analysis

#### Cell morphology analysis.

Swiss 3T3-J-2 cells are seeded in a 96-well plate with 10,000 cells per well and cultured for 48h at 37 °C. Here, the cells are exposed to pure PNIPAM hydrogel, AgNWs/PI nanocomposite, RGO/PI nanocomposite, PEDOT:PSS/PI nanocomposite, MXene/PI nanocomposite, and the integrated soft robot. Notably, each of these functional units was encapsulated with a parylene film (thickness ~ 2 μm). For examining the effect of thermal stimulation on cell viability, the 3T3-J-2 cells with a soft robot are subjected to an environment at 39 °C for a duration of 48h. To detect Vimentin antigen, chromogenic Immunohistochemistry (IHC) is performed on paraffin-embedded cells that were sectioned at 5 microns. This IHC is carried out using the Leica Bond Rx Autostainer system. Slides are dewaxed in Bond Dewax solution (AR9222) and hydrated in Bond Wash solution (AR9590). Heat-induced antigen retrieval is performed at 100°C in Bond-Epitope Retrieval solution 1 pH-6.0 (AR9961). After pretreatment, slides are incubated with Vimentin Antibody (5741, Cell Signaling Technologies) at 1:1000 for 30m followed with Novolink Polymer (RE7260-CE) secondary. Antibody detection with 3,3’-diaminobenzidine (DAB) is performed using the Bond Intense R detection system (DS9263). Stained slides are dehydrated and coverslipped with Cytoseal 60 (8310–4, Thermo Fisher Scientific). A positive control tissue is included for this run. High-resolution acquisition of IF slides is performed with the Aperio Versa 200 scanner (Leica Biosystems Inc.) at an apparent magnification of 20X. Immunofluorescence reaction (IF) is performed on paraffin-embedded cells that are sectioned at 5 microns. This assay is carried out on the Bond Rx fully automated slide staining system (Leica Biosystems) using the Bond Research Detection kit (DS9455). Slides are dewaxed in Bond Dewax solution (AR9222) and hydrated in Bond Wash solution (AR9590). Heat induced antigen retrieval is performed at 100°C in Bond-Epitope Retrieval solution 1 pH-6.0 (AR9961) for 30 minutes. After pretreatment, slides are incubated with Vimentin Antibody (5741, Cell Signaling Technologies) at 1:1000 for 30m. Ready-to use secondary antibody, Leica’s Novolink Polymer (RE7260-CE) is used followed by TSA Cy5 (SAT705A001EA, Akoya Biosciences) to visualize the target of interest. Nuclei were stained with Hoechst 33258 (Invitrogen). The stained slides are mounted with ProLong Gold antifade reagent (P36930, Life Technologies). Positive controls are included for each assay. High resolution acquisition of IF slides is performed with the Aperio Versa 200 scanner (Leica Biosystems Inc.) at an apparent magnification of 20X.

#### Cell viability test

Swiss 3T3-J-2 cells are seeded in a 96-well plate with 10,000 cells per well and cultured for 48h. Here, the cells are exposed to pure PNIPAM hydrogel, and the integrated soft robot. For examining the effect of thermal stimulation on cell viability, the 3T3-J-2 cells with a soft robot are subjected to an environment at 39 °C for a duration of 48h. We use the Aperio Cytoplasmic version 2 algorithm on image regions that were annotated to exclude artifacts. The algorithm input parameters included Clear Area Intensity, Optical Density values (RGB) for both counterstain and biomarker detection, intensity threshold values, and minimum/maximum size and smoothing values for cell segmentation. Several of these parameters were adjusted to apply to the specific marker (Vimentin) and cells.

#### Histological analysis

Explanted organs were bisected and stored in 10% buffered formalin inside 50 mL conical tubes. Following this, these tissue samples were subsequently prepared for histological evaluation with H&E staining and were imaged using Leica Biosystems.

### A soft robotic thera-gripper for epicardial sensing and pacing

#### The fabrication of a robotic thera-gripper

The e-skin layer of the soft robotic gripper is formed using the laser cutting machine, consisting of four strain sensors made of serpentine Au/PI resistors, two Au pacing electrodes, and two temperature sensors made of thermal resistors, as shown in **Fig. S52A&B**. Following the application of a parylene film, four pieces of PNIPAM hydrogel were bonded onto the e-skin layer to form the actuators. The soft robotic gripper can gently hold a mouse heart during its beating movement.

#### In vivo animal experiment.

Procedures used in the study were reviewed and approved by the Institutional Animal Care and Use Committee and Research Animal Resources at the North Carolina University Chapel Hill (IACUC ID:21–241.0). Female mice (weight 20 to 30 g; age, 10 weeks) were purchased from the Jackson Laboratory. The detailed surgery process is described in **Supplementary Note S9**. The electrocardiography (ECG) and heart function were monitored simultaneously using commercial equipment (PowerLab). The myocardial infarction surgery was conducted by permanently occluding the left coronary artery (LCA). Echocardiography was performed to evaluate cardiac function and histological analysis was performed to assess inflammatory effect of the implantable robotic thera-gripper.

## Data Availability

All data needed to evaluate the conclusions in the manuscript are present in the manuscript and/or the Supplementary Materials.
